# Flourishing within vulnerability: on human fragility and the conditions for a habitable environment

**DOI:** 10.1007/s11017-026-09764-3

**Published:** 2026-06-24

**Authors:** Irene Quiliconi

**Affiliations:** Department of Humanities, University of Ferrara, Ferrara, Italy

**Keywords:** Alasdair macintyre, Applied phenomenology, Flourishing, Lived illness, Vulnerability, Well-being

## Abstract

In recent philosophical discourse, flourishing has emerged as a concept of growing importance—often linked to the field of well-being studies and explored by various and different disciplines. The article approaches flourishing not as an abstract ideal, but rather as a condition that must be understood as rooted in the lived reality of human vulnerability. More specifically, I argue that any genuine account of human flourishing must regard vulnerability— human inherent fragility and reciprocal dependence—not as an impediment, but as the very foundation from which meaningful flourishing can arise. Building on Alasdair MacIntyre’s reflections—who observes that this perspective is insufficiently acknowledged in comparison to the prevailing standard of moral philosophy—I understand vulnerability as a fundamental human characteristic arising from the interconnectedness of dependence, rationality, and animality. The second part of the paper engages with phenomenological perspectives in order to establish a dialogue between this tradition and MacIntyre’s framework of virtue ethics. The aim is to anchor the concept of a virtuous moral life—implicit in the notion of flourishing—in the lived, embodied experience of the individual. In this light, three fundamental categories intrinsic to human vulnerability are identified as essential compass points for understanding what must be preserved, pursued, and guaranteed even under conditions of extreme fragility. Only by attending to these dimensions—embodiment, co-existence and lived temporality—can ensure that the human environment remains habitable, and thus adequate for a disposition toward flourishing.

## Introduction

The concept of *flourishing* is perhaps nowadays more pervasive than ever, having reached a significant regard in everyday life. From a general and socially widespread point of view, flourishing is intended as a life well-lived, where one feels to have the possibility of expressing one’s own specific capacities and desires, thus fostering a certain overall degree of life satisfaction. Accordingly, it is not surprising that, at both social and cultural levels, there is growing attention toward self-care and the potential attainment of such a state, as well as to the means and processes through which it may be realized.^1^

Talking about flourishing can easily recall the familiar concept of *well-being*. Especially in everyday life the two terms are often used interchangeably, insofar they undeniably share some common features. Nevertheless, the two terms are not equals, and their conceptual foundation diverge across disciplines. In the psychological tradition, human well-being is conceptualized as a positive state involving various spheres^2^ – such as physical and mental health, emotional balance, social life—marked by the harmonious satisfaction of both objective and subjective needs [2].

The term flourishing has been largely used in philosophical studies as the privileged way to translate the Greek notion of *eudaimonia,* intended as the highest human good. As widely acknowledged, particularly referring to Aristotelian Ethics, *eudaimonia* has a much deeper meaning than that of “happiness”. It denotes an overall well-lived life, within which virtues are cultivated and expressed through intentional activity that foster human specific capacities [3]. Grounded in Aristotelian thinking, this perspective has found particular expression in the philosophical tradition referred to as virtue ethics [4–6]. As a translation of the Greek term *eudaimonia*, the notion of flourishing conveys a deeper and more comprehensive dimension of human life than the concept of well-being alone. While it may include aspects typically associated with well-being, such as personal satisfaction and balance, flourishing is fundamentally rooted in an *ethical* framework. It emphasizes the overall meaning and ethical direction of one’s life, shaped by virtuous action, deliberate choice, and the cultivation of human full potential in accordance with reason. It is in this respect that flourishing engages with a deeper existential dimension, as it entails one’s sense of identity and the aspiration to live in accordance with who one genuinely believes oneself to be—or ought to become.^3^

Hence, although the terms *flourishing* and *well-being* are not fully synonymous, they are certainly related and, in many respects, complementary. Similarly, while their conceptual foundations and disciplinary contexts—philosophical and psychological—differ, scholars have increasingly emphasized the value of integrating these perspectives [9]. This convergence aims to offer a more comprehensive understanding of human development, one that bridges ethical reflection with empirical insights into individual and collective well-being.

From this initial terminological exploration, it should appear clear that the notion of flourishing seems to encompass, or at least go beyond, that of well-being. If the two concepts are different, and have been differently investigated, a fundamental question arises: is it possible to flourish even when some of the objective features considered essential to well-being are missing? For example, can one still flourish when psychological or physical well-being is compromised? Since, drawing from both historical and philosophical traditions, the answer appears to be affirmative, one needs to understand how it is possible, and what are the implications of this assumption.^4^

## Human constitutional vulnerability

As mentioned above, the philosophical perspective of virtue ethics focuses on the overall ethical trajectory of an agent’s life, that is, on the chosen repeated actions and passions through which virtuous—or vicious—*hexeis* are gradually developed. Within this framework, however, an element that cannot be overlooked is, as Martha Nussbaum suggests, *fortune* [12]*.* Indeed, fortune plays a significant ethical role insofar as it provides—or withholds—the basic conditions that shape a person’s general well-being and capacity for action. Clearly, external and unpredictable factors influence the extent to which individuals are able to develop their human potential and pursue their aspirations. Human beings are inevitably exposed to fortune. Various contingent circumstances affect our physical and mental states as well as our broader life conditions. Consider, for example, a previously healthy individual who is suddenly struck by a severe illness. This person’s situation changes dramatically due to forces entirely beyond their control. In such circumstances, essential aspects of what one ordinarily conceives as well-being may be significantly compromised or even disrupted. To echo the previous question^5^: is it possible to remain virtuous and to flourish despite such adverse conditions brought about by fortune?

Addressing this question leads to a more fundamental reflection on the human condition itself. While fortune refers to external circumstances that may affect one unpredictably, and to which humans are inevitably vulnerable, *vulnerability *per se points to a foundational level: it is an intrinsic and constitutional feature of human existence. Many philosophers have emphasized that vulnerability is not merely an occasional condition affecting particular individuals, but rather a universal dimension of human life.^6^ If vulnerability is indeed an intrinsic aspect of the human condition, then any reflection on human flourishing would remain incomplete without taking it into account. At this point, however, an important distinction must be made. The fact that vulnerability belongs to the human condition does not by itself imply that it should be regarded as valuable. One might instead view vulnerability simply as an unfortunate limitation on human agency. Yet a different interpretation emerges when vulnerability is considered in relation to the development of human rationality and moral life. Certain forms of vulnerability—particularly those connected with relations of dependence—play a formative role in shaping the capacities through which individuals come to deliberate, act, and pursue shared goods. In this sense, vulnerability may be understood not only as a constraint but also as a condition that makes possible a distinctively human form of flourishing.

This perspective finds significant support in the thought of Alasdair MacIntyre, particularly in his reinterpretation of virtue ethics in light of a contemporary framework that emphasizes the inherent vulnerability of human beings and their susceptibility to various forms of affliction [16].

## Dependence, rationality and animality: MacIntyre’s account of vulnerability

Building on the foundational insight that human vulnerability is intrinsic and universal, MacIntyre further develops these ideas by examining how dependence, rationality, and animality intersect in the moral life, highlighting the relational and embodied conditions necessary for flourishing.

In the opening chapter of *Dependent Rational Animals*^7^, MacIntyre challenges the dominant philosophical narrative that prioritizes autonomy, independence, and rational self-sufficiency. Instead, he argues that *dependence* on “particular others” is not an accidental or temporary feature of human life, but rather a fundamental and enduring aspect of the human condition. The recognition of this dependence has long been emphasized within feminist philosophy, particularly through the development of care ethics. Scholars such as Eva Kittay [17] and Gail Weiss [18] have highlighted that all individuals exist within networks of mutual reliance, and that caregiving, vulnerability, and relational interdependence are central to ethical, social, and political life. Kittay, for example, underscores the societal importance of supporting caregivers and recognizing the role of the “dependency worker,” while Weiss emphasizes the intercorporeal, relational nature of human embodiment. MacIntyre, in turn, situates his account of dependence and vulnerability within this broader discourse, noting that while these contributions are significant, “such work is only the beginning to make any systematic impact on what is currently established as mainstream moral philosophy in the advanced societies of the West” [16, p. 4].

This insight calls into question the widespread tendency to treat individuals who are ill, injured, or disabled as belonging to a separate category—them—conceived as fundamentally different from *us*, the supposedly rational, healthy, and autonomous individuals. Such a dichotomy obscures the fact that vulnerability is a dimension of every human life. It characterizes early stages of development, emerges again in moments of illness or crisis, and often becomes more visible in later stages of life. The recognition of this shared vulnerability therefore challenges the assumption that rational agency can be understood independently from relations of dependence and care.

MacIntyre observes that what he calls “mainstream moral philosophy” has largely failed to acknowledge this reality in two respects. First, because it often assumes that full humanity is realized only through independence and rational self-sufficiency, thereby neglecting the constitutive role of interdependence and cooperation. Second, it relies on an understanding of rationality that is detached from the embodied nature of human life, as if reason could function independently of our physical and biological existence. Against this view, MacIntyre proposes a different understanding of the human being as a rational animal whose rational capacities develop within embodied and relational forms of life. MacIntyre therefore raises a set of questions that shift the focus of moral philosophy toward the reality of human dependence: “What difference would it make if we were to treat the facts of vulnerability and affliction, and the related facts of dependence, as central to the human condition? And how should we begin to try to answer this question?” [16, p. 4]. These questions provide an important starting point for reconsidering the relationship between vulnerability, dependence, and human flourishing.

Human vulnerability, far from being an accidental limitation, is intimately connected to the fact that rationality itself emerges within networks of care and cooperation. Dependence, rationality, and animality must therefore be understood in relation to one another.

Vulnerability is, by its very nature, a *relational* category, just as the human being is a relational creature. In response to the second assumption of standard moral philosophy, MacIntyre seeks to rethink the relationship between our animality and our rationality, seeing this integration as a distinctive feature of the human animal species. This leads to a reconsideration of the constitutive connection between human bodily existence, in which human animality is primarily expressed, and human vulnerabilities. Equally significant is the fact that it is through one’s bodily existence—and therefore through one’s “animal identity”—that one engages in the most immediate and primary form of relation with others. If “dependence, rationality, and animality have to be understood in relationship to each other” [16, p. 5], then what kinds of virtues are required to ensure a meaningful and authentic form of human flourishing? MacIntyre argues that what is essential is the integration of two complementary sets of virtues: the “virtues of independent rational agency”—such as justice, courage, and practical wisdom—and the “virtues of acknowledged dependence”—such as trust, compassion, patience, and care. Indeed, these are not opposing alternatives, rather, they are mutually supportive dimensions of a flourishing human life.^8^

A clear illustration of this dynamic can be found in MacIntyre’s discussion of child development [16, pp. 81–90]. The development of rational agency does not occur in isolation but within networks of care, education, and relational support. Children gradually acquire the capacity for independent reasoning through relationships in which they initially depend on others for guidance, protection, and recognition. In this sense, autonomy itself emerges from prior relations of dependence. Drawing on the work of Donald Winnicott, MacIntyre therefore emphasizes that the acknowledgment of dependence is a crucial condition for the development of genuine independence. [16, 19].

Even if vulnerability is a constitutive feature of the human condition—one that is always present, even when one does not consciously perceive oneself in those terms—it is undeniable that certain stages of life are more visibly marked by it. Consider childhood and old age, notably fragile. In addition to these, there are also specific moments—or more enduring conditions—of tangible suffering, whether physical, psychological, social, or economic, in which one’s dependence on others becomes more evident and our sense of fragility more acute. Such experiences of affliction, to which one is perpetually exposed, may thus serve as moments of existential unveiling, disclosing a profound truth about what it means to be human. If suffering—broadly conceived—has the power to reveal certain aspects of one’s human identity, it must be emphasized that this revelation occurs primarily within a *relational* dimension: one that shows how one’s life is fundamentally embedded in networks of relationships [20]. Re-engaging with MacIntyre’s argument, one is reminded that one is dependent upon “particular others” from whom one requires care, special attention, and recognition. This dependence is not incidental but constitutes a central feature of moral and social life.

From this perspective, human flourishing can only occur within a relational and plural space in which the virtues of independent rational agency are harmonized with those that arise from the acknowledgment of our mutual interdependence. Because human rationality and moral agency develop within relations of dependence, vulnerability plays a formative role in shaping both one’s practical reasoning and our moral identity. The need for care, trust, and cooperation provides the context in which individuals learn to recognize the needs of others, to deliberate about shared goods, and to cultivate virtues that would otherwise remain unintelligible. In this sense, certain forms of vulnerability are not simply obstacles to flourishing but conditions that make possible the development of relational virtues and the moral capacities necessary for a fully human life. The richness of human potential is not diminished by vulnerability; rather, it is grounded in it.

Connecting with the subtitle of his book—*Why Human Beings Need the Virtues*—MacIntyre contends that virtues are indispensable for achieving the human *telos*, or highest good, and thus for genuine flourishing. Far from being traits of merely individual excellence, virtues function as moral capacities that enable us to navigate the conditions of vulnerability and interdependence that characterize human life. They are essential for the formation of just and supportive social relationships, which in turn make the pursuit of the good life possible.

## Phenomenological insights

Building on MacIntyre’s account of vulnerability, it can be argued that flourishing remains possible even under conditions of suffering, or when certain features traditionally associated with well-being are absent. If human beings are inherently vulnerable and dependent on one another, then this condition is not an exception but a defining characteristic of human nature. While this frailty may manifest more visibly in some individuals, it is a universal feature of the human condition. If one denies the possibility of flourishing to those we categorize as ill, disabled, or otherwise weaker, what is one truly implying? Is one not also denying the potential for flourishing in oneself, given that one too is—whether one admits it or not—vulnerable and dependent in various ways? If vulnerability is used as a reason to exclude others from the possibility of flourishing, then that same standard could ultimately be used to exclude everyone.

Theoretically, it should be quite easy to agree with these claims. Clearly, asserting them from a practical point of view is much more difficult.

At this point, it is useful to consider how phenomenology can contribute to understanding flourishing in the context of vulnerability. The guiding questions are the following: what contribution can the phenomenological approach, broadly conceived, offer to this line of reflection? More specifically, how might it be helpful to situate virtue ethics’ characteristic focus on the *hexis* of the agent and virtuous life within a phenomenological–existential framework?

Phenomenology offers two interrelated contributions. First, it provides a descriptive, experiential insight into how vulnerability is lived. This highlights the situated, embodied, and relational character of human life showing that vulnerability is not merely an occasional limitation but a constitutive aspect of human existence. Second, phenomenology suggests normative implications when interpreted alongside virtue ethics: by showing how vulnerability shapes experience, it indicates which capacities and virtues —such as attentiveness, care, openness, and responsiveness— are necessary for human flourishing in a relational and fragile world, without itself prescribing norms. In the existential-phenomenological tradition, this is closely tied to a distinctive conception of human existence as being-in-the-world, as always already situated within a specific context. This approach has proven fruitful in medical humanities and clinical practice, where it has been recognized as a valuable framework for attending to patients’ embodied vulnerability, subjective suffering, and lived experience of illness. In such contexts, the phenomenological emphasis on situatedness and relationality has offered a powerful alternative to reductive biomedical models, thus reinforcing the relevance and adequacy of phenomenological reflection in addressing human vulnerability.^9^

## The *lived* experience of illness

A substantial body of work has developed within phenomenology to explore the lived experience of illness and its implications for understanding well-being and embodiment.^10^ On this account, the phenomenologist S. Kay Toombs offers particularly compelling insights, informed by her own severe neurodegenerative illness. Toombs emphasizes the first-person, subjective experience of illness [25], showing how being ill profoundly transforms one’s understanding of well-being 11and the self. Illness is experienced not merely as a biological condition to be treated, but as a distinct way of being-in-the-world. Toombs stresses the significance of the body—not merely as the site of physical suffering, but as the very medium through which suffering is experienced existentially. *My body* is my particular and unique point of view on the world. It is through my body and its relationship to spatiality that certain possibilities are opened up, while others are withheld. I place myself into the world through and thanks to my body. Most importantly, Toombs reminds us of what Merleau-Ponty suggestively argued, “I do not only have a body; I *am* my body” [25, 26]. This means that any disruption of the body also entails a disruption of the self, with a consequent profound psychological and existential impact. Spatiality is inevitably imbued with existential significance, as it is not merely an objective or measurable dimension but is lived, experienced, and interpreted by embodied subjects situated in the world.

What is most relevant for the present reflection is the further point that Toombs adds:

“illness—experienced as a wounded body—is like coming face-to-face with one’s inherent vulnerability and dependence upon one’s body” [25, p. 73]. Considering MacIntyre’s argument, this may be understood as the full disclosure of human *animality*. Indeed, similarly to Toombs, he argues that “human identity is primarily, even if not only, bodily and therefore animal identity and it is by reference to that identity that the continuities of our relationships to others are partly defined” [16, p. 8]. The disruption of bodily intentionality profoundly alters one’s being-in-the-world, beginning, for instance, with basic limitations on the ability to move through space. If human relationships are fundamentally grounded in bodily encounters, a disruption of one’s own body will inevitably bring about significant changes. Within the experience of sufferance, human dependence becomes more evident than ever—and in the case of severe illness, it is undeniably heightened.

Toombs also draws attention to the profound transformation which is almost always present in one’s relationship with temporality that occurs in conditions of severe illness. The disruption of the body—and the consequent disruption of the self—alters one’s proper and unique way of being in the world, requiring a shift toward a new pattern of orientation within it. This dynamic involves not only spatiality but also temporality, both understood as existential and lived dimensions. Thus, as with spatiality, what matters is not the objective temporality measured by clocks, but the subjectively lived temporality, which is enriched with unique meaning by each individual. Toombs denounces the painful experience, brought about by illness, of a temporal disruption in her overall personal lived experience. In this context, and reflecting on the relationship between the ill person and the physician or other close contacts, Toombs supports the idea of a *narratively* focused approach to the patient—one that values the subject’s unique story as lived from the first-person perspective. Narration serves the crucial function of meaningfully acknowledging the patient’s unique “biographical situation” 12[25, p. 5], understood as a world that is spatially and temporally organized around the patient, who is positioned at the very center of this experiential unfolding. It is particularly noteworthy that, at this point in her argument [25, p. 6], Toombs explicitly cites MacIntyre—thereby confirming and reinforcing the continuity I have been outlining so far. MacIntyre [28] affirms that the temporal structure in which one lives is exhibited in a “present-now,” which is “always experienced within the horizons of past and future” [25]. This dynamic is referred to as a “life narrative,” and it is precisely this kind of existential experience that Toombs sees as being mirrored in the practice of clinical narrative. This practice is essential in giving the patient the possibility of re-establishing a temporal coherence in their life, thus countering the sense of loss of integrity and meaning.

From Toombs’ phenomenological perspective, while primarily experiential, one can deduce some implications valuable for virtue ethics. Observing how vulnerability manifests in lived experience may indicate which virtues and moral capacities are required to support human flourishing under such conditions. Phenomenology, in other words, grounds ethical reflection in the realities of embodied and relational vulnerability, showing that flourishing is intimately connected to embodied vulnerability and relational dependence. The subjective persistence of well-being, even amid profound suffering as Toombs documents, demonstrates that flourishing is not precluded by vulnerability but rather is realized within it. Integrating these insights, virtue ethics gains a more nuanced understanding of moral development, emphasizing that the cultivation of virtues must occur in dialogue with the lived realities of fragility, dependence, and relational engagement. More specifically, the idea is that phenomenology may offer heuristic tools for developing a basic existential framework within which to situate reflection on the overall trajectory of an agent’s moral life as approached by virtue ethics. In this regard, engaging with MacIntyre’s position—among the many authors in the virtue ethics tradition—is not at all undeliberately chosen. Indeed, he provides a robust normative framework for practical rationality and the formation of virtuous character, while placing great emphasis on vulnerability as a constitutively situated condition of human fragility.

Toombs’ account further illustrates that objective conditions traditionally associated with well-being are on their own insufficient to a genuinely perceived state of well-being: subjective and existential dimensions are essential. Even under severe illness, well-being can persist through readjustment to one’s being-in-the-world [25]. Therefore, the possibility of flourishing is not negated by vulnerability.

Clearly, acknowledging the subjective dimension and perception of well-being does not mean that everything collapses into basic relativism. The virtues that human beings are capable of cultivating — and which shape the possibility of flourishing — rest on objective grounds.

Reflecting on what appear to be the essential and commonly shared conditions for well-being and human flourishing becomes particularly important in situations where there is a need to establish institutional frameworks or provide some form of social and state-level assurance for well-being — especially under difficult or extreme circumstances. While well-being is undoubtedly shaped by subjective factors, identifying and acknowledging the generally shared conditions that make it possible is crucial when such reflection informs public policies or institutional practices. One such example is the field of palliative care. Thus, if we agree that well-being and flourishing are possible even under conditions in which our constitutional vulnerability is expressed at its highest level, a further question arises: what foundational conditions—beyond the mere provision of medical care or resources—should we seek to guarantee in order to make flourishing possible in those very situations?

Answering this question requires a perspective that goes beyond technical or institutional provision. It must attend also to the lived experiences of patients, the relational contexts in which they are embedded, and the temporal structures that shape their perception of life’s coherence and meaning.

## A humanly* habitable* environment

Moving toward a broader existential perspective, the notion of a “humanly habitable environment”, as developed by the phenomenologist Jean Ladrière [29], becomes particularly instructive. By focusing on embodiment, co-existence, and lived temporality, Ladrière provides a framework for understanding how an environment can genuinely support human flourishing.

The aim is to develop a theoretical framework that can guide the operationalization of the concept of well-being in clinical contexts, such as that of palliative care. The reason why embodiment, interrelations and lived temporality may serve as guiding criteria for this line of reflection lies in a simple fact: they are intrinsically grounded in the reality of our human finitude and vulnerability. These dimensions are present both in conditions of ‘ordinary’ vulnerability, shared by all, and in conditions of extreme fragility. Recognizing this continuity is crucial to avoid a preconceptual separation between the vulnerable—MacIntyre’s “them”—and others conceived as strong, independent, and healthy human beings [16].

The existential dimensions considered by Ladrière closely resonate with MacIntyre’s account of human dependance and vulnerability, providing a conceptual bridge from descriptive insights into lived vulnerability. Briefly consider how Ladrière accounts for these three existential conditions – comparing them with MacIntyre’s reflection, as shown in Table 1.

**Table 1:** Comparison of the three existential categories between MacIntyre and Ladrière

	Virtue ethics (MacIntyre)	Phenomenology (Ladrière)
Embodiment	Human animality: having a body	Lived body (*Leib*), embedded experience
Relations	Human constitutive interdependence	Body situated in a field of interaction: co-existence
Temporality	Narrative subject; development of virtuous *hexeis*	Lived temporality; one own meaningful life-history

It is through the body that an existent situates themselves in the world. If the body serves as a mediator between existence and the world, it does so only insofar as it is always already situated within a shared space of interaction – so that one has the second existential condition of co-existence. This certainly resonates with MacIntyre’s account of the animal identity of human beings, grounded on the fact that humans have bodies – and intended as the first source of interrelation. And existence—understood both in terms of corporeality and co-existence—is, in its essence, movement. This ontological condition finds its concrete expression in the structure of lived temporality — a temporality that is never abstract, but deeply situated and embodied. As comparison with MacIntyre, recall what also Kay Toombs emphasizes: the idea of a series of present-moments, that shape the subject’s life as narrative life. The narrative dimension also seems to coincide with the temporal dimension within which it is possible to develop virtuous *hexeis*.

In Ladriere’s framework, ethical life and the pursuit of existential fulfillment—which entails an openness to a deep meaningful dimension—manifest themselves through actions that are oriented toward a certain ‘good’. Ladrière places particular emphasis on the criteria of embodiment, co-existence and lived temporality not as normative principles meant to directly guide ethical action. Rather, they are *foundational* conditions without which human action cannot unfold authentically. Actions, though always particular and singular in their occurrences, acquire meaning within the broader horizon of the agent’s entire existence. In this sense, Ladrière’s account echoes virtue ethics’ concern with the formation of character through repeated actions, but with one difference. Rather than articulating moral norms, Ladrière brings us back to the *pre-ethical* level, to the structural conditions of human experience, where ethics is not yet codified, but is being prepared and takes shape.

By attending to these fundamental conditions, Ladrière’s framework allows one to identify the minimal yet indispensable criteria that make a space truly “humanly habitable”—one in which ethical action, relational engagement, and the pursuit of a meaningful life can find their proper ground even amidst extreme fragility. A humanly habitable environment is one that the situated subject perceives as appropriate or fitting, and with which one maintains a relationship of harmony and mutual exchange. The core assumption of Ladrière’s argument is that one cannot act ethically in a world that is not, first and foremost, habitable.

Despite the differing approaches of the two authors, Ladrière’s categories offer, in an existential and phenomenological key, a kind of translation of what MacIntyre considers essential for human flourishing: the need for a shared space in which human vulnerability and dependence are not denied, but rather recognized and affirmed. Grounded in the finite, limited, and fragile nature of human existence, Ladrière’s notion of a humanly habitable environment complements and extends the ethical analysis, showing that flourishing is not only about achieving a positive state or exercising autonomy, but also about cultivating spaces in which individuals can develop agency, maintain meaningful relationships, and experience their lives as coherent and ethically resonant.

## Where theory meets care

Human flourishing is not the exclusive domain of autonomy, strength, or full rational capacity. It remains possible—even in situations of extreme vulnerability—provided that certain foundational conditions are preserved. Indeed, it is precisely under such conditions that the question of meaning and ethical integrity becomes most pressing.

This convergence between Ladrière’s phenomenological insights and MacIntyre’s virtue ethics offers a fertile foundation for rethinking ethical care—not only in theory, but in practice. A particularly relevant example can be found, as already mentioned, in the context of palliative care, where the expression of human vulnerability reaches one of its most radical forms, and where bureaucracy and medical protocols must coexist with existential needs. In these contexts, the signaled philosophical convergence may help one imagine new ways to make space for flourishing. It may help one to see the dimension of time as something limited but nevertheless precious and still existentially open: open to meaning, to relationship, to the world. In this perspective, interdependence becomes a vital force, capable of sustaining strong and responsive social networks. When properly structured, such networks provide not only support but also the existential space in which the virtues can be cultivated, and the conditions for flourishing can be meaningfully preserved.

To see how these philosophical insights can be translated into concrete practice, it is helpful to consider illustrative scenarios in which attention to embodiment, relationality, and temporality can make a tangible difference in patient care. For instance, consider a patient in a hospice setting whose physical condition severely limits mobility and autonomy. While medical staff focus on symptom management, a phenomenologically informed approach attends to the patient’s lived experience: helping them maintain meaningful relationships, engage in personally significant activities, and exercise agency in daily routines. In some cases, such attentiveness allows patients to experience moments of flourishing—sharing stories with loved ones, participating in family rituals, or finding satisfaction in small acts of creativity—despite the backdrop of severe illness. Conversely, environments that emphasize procedure over presence can leave patients feeling isolated and depersonalized, illustrating the practical importance of merging virtue ethics with phenomenological attention to embodiment and relationality [22, 23, 30].

Increasingly, patients express not only the desire to die with dignity, but also the desire to *live* their end of life within a space that feels familiar and personal [31–33]— an environment they perceive as their own.^13^ This desire reflects a deeper existential need: the preservation of a minimal sense of bodily integrity, of genuine and meaningful relationships, and of a narrative that grants coherence to one’s life, even as it nears its end. Thus, the need is towards spaces that are not only and mainly clinically appropriate, but also *existentially* meaningful: in short, and in Ladrière’s terms, humanly habitable.^14^

In this very respect, the three categories proposed by Ladrière—embodiment, coexistence, and lived temporality—may prove to be valuable heuristic criteria for assessing whether a given environment can truly support human presence in its fullness. They offer a phenomenological framework through which abstract ideals such as flourishing and well-being can begin to be translated into the concrete realities of clinical and institutional practice. More specifically, they allow one to ask: does this environment support the lived body? Does it sustain human connection? Does it allow time to be experienced meaningfully — not simply measured, but lived? In this light, flourishing becomes not an abstract goal, but a lived possibility—one that requires the active construction of environments open to meaning, capable of sustaining relationships, and respectful of human fragility.

Flourishing, then, even at the threshold of life, becomes conceivable not despite fragility, but precisely *through* it—when fragility is met with care, recognition, and a world that remains open and habitable.

## Conclusions

I have argued that to address vulnerability in the proper way, one must understand its being rooted in human nature. As MacIntyre argues, it would not otherwise be possible to cultivate human virtues and to obtain a fully human sense of flourishing. The interaction between phenomenology and virtue ethics that I have proposed entails a mutual enrichment: phenomenology provides heuristic tools to grasp the lived experience and embodied dimension of moral agency, while MacIntyre’s virtue ethics offers a normative and narrative framework that situates such experience within a tradition and a life project oriented toward character formation. This synergy allows for an integration of the normative and the existential dimensions, highlighting vulnerability and fragility as fundamental aspects of concretely situated moral agency. My hope is that deeper investigations on the fructuous intersections between the two approaches will be carried out, with the goal of applying them to situated, concrete and urgent everyday scenarios of human vulnerability.

In medical and fragile horizons like that of palliative care, where protocols often risk overshadowing the patient’s specific presence, questioning what kind of existential spaces we are building becomes an urgent question. Acknowledging the vulnerability that link us all, we should gain more promptness in addressing the existential demands emerging from these situations – with a more conscious sense of mutual responsibility. Reflecting on the categories of embodiment, co-existence and temporality has not been done for the purpose of a mere theoretical exercise. The goal is to obtain a concrete and situated knowledge of our intrinsic vulnerability, in order to understand how to move within it and how to follow the path for a sense of flourishing. The case of the palliative care certainly is a delicate stage within one’s life, and it needs to be addressed by a broad approach – both on the medical and on the existential level. Reflecting on the human habitability of the environments in which these paths are carried, means to ask if they truly are capable of sustaining life in its fullest, most vulnerable, most human, form.

## Footnotes

1 An interesting case is the great variety of podcasts that deal with the theme of flourishing, with matching frequency on television and newspapers. To make just an example, BBC Radio 4’s program *All in the Mind* has chosen *Flourishing* as one of its main themes, making the case for a growing attention on the concept and its difference from well-being. More specifically, the interviewer discusses with the psychologist Dr Peter Olusoga about the results of the *Global Flourishing Study*—a huge new study involving more than 200,000 people in 22 countries. For more information on the study see the website https://globalflourishingstudy.com/

2 Carol D. Ryff proposes a six-factor model of psychological well-being that includes self-acceptance, positive relationships, autonomy, environmental mastery, purpose in life, and personal growth. This framework has become one of the most influential models in the study of well-being [1].

3 In healthcare contexts, both the concepts of “quality of life” and “well-being” incorporate subjective and multidimensional elements. Particularly in palliative care, quality of life assessments includes patients’ self-reports across physical, emotional, social, and functional domains, reflecting their lived experience of illness and treatment. Well-being, however, is typically conceived as a broader construct that embraces these dimensions while also involving psychological and existential factors such as meaning, coherence, and relationality. Importantly, flourishing (or eudaimonia) goes further by emphasizing a normative ethical dimension: it involves realizing one’s human potential and living a life of virtue, integrity, and authentic agency. While well-being describes a state or experience that can be assessed and reported, flourishing entails the active pursuit and actualization of human goods in a morally coherent way. This distinction is particularly relevant in clinical and end-of-life contexts, where patients may experience flourishing—characterized by dignity, relational depth, and existential meaning—even when quality of life scores or conventional indicators of well-being are low. For a further discussion, see: Nussbaum [7] on flourishing as the realization of human capabilities and virtue, and [8] for recent debates on subjective quality of life in palliative care.

4 An insightful example may be that of the biblical figure of Job, protagonist of *The Book of Job*. Indeed, the figure of Job has been examined as a paradigmatic case of moral integrity and virtue under extreme suffering. Vesely [10] compares Job’s steadfast righteousness with Aristotelian *eudaimonia*, suggesting that the narrative portrays flourishing as grounded in moral character rather than material or physical well-being. As Aristotle himself notes (e.g., in his discussion of Priam in *Nicomachean Ethics*, Book I), extreme misfortune can, in some cases, limit the full realization of a virtuous life, though virtue enables the individual to better navigate ordinary turns of fortune. Thus, flourishing may be constrained under extraordinary adversity, but moral agency remains operative. Important case studies in recent years explore how flourishing may remain possible even under significant psychological distress. For example, Neijzen [11] examines how individuals experiencing depression may struggle to recognize and respond to opportunities for virtuous action. The study concludes that while such conditions may impede moral agency, they do not entirely preclude the possibility of flourishing.

5 The seemingly differing formulations of the key question are not intended to be contradictory. The first question highlights that flourishing can occur even when some objective features typically associated with well-being are absent. The second introduces the influence of fortune, showing how external circumstances may disrupt these features. Finally, the discussion of constitutional vulnerability emphasizes that certain aspects of human fragility are intrinsic, grounding flourishing in the fundamental conditions of embodied, relational, and temporal existence.

6 To give just a brief insight on the diverse approaches and fields in which vulnerability has been considered: see Nussbaum [7, 13], who frames it as a basis for justice grounded in shared human needs; Butler [14], who highlights vulnerability as the foundation of interdependence and political resistance; Levinas [15], for whom vulnerability is the ethical opening to the Other.

7 In the *Preface* of the book, MacIntyre writes: “But the philosopher whose failure to recognize the importance of these questions and whose consequent errors and limitations I am chiefly anxious to correct is myself” [16, p. IX]. Thus, he explicitly acknowledges a major shift in his ethical thinking, revising positions from *After Virtue*, *Whose Justice? Which Rationality?*, and *Three Rival Versions of Moral Enquiry*. He admits the earlier error of attempting to separate ethics from biology, now arguing that any adequate account of virtues must be rooted in our embodied, vulnerable, and animal nature. This marks a significant turn toward a more biologically and relationally grounded conception of human flourishing.

8 Here, I do not follow more closely MacIntyre’s account of the two sets of virtues. To understand what MacIntyre really means for “acknowledged dependence virtues” refer to [16, pp. 119–128]. The most important point he makes about this topic is the fact that virtues of dependency intrinsically comprehend both aspects of giving and receiving.

9 For a critique consideration of the influence that the phenomenological approach has gained within medical studies, see [21].

10 As shown, for instance, in the works by Havi Carel [22], Drew Leder [23] and Fredrik Svenaeus [24].

11 If we recall the previously mentioned conception of well-being as a combination of both objective and subjective dimensions, it becomes clear that the latter holds primary importance in this context.

12 Toombs takes the idea of the individual’s unique biographical situation from the phenomenologist Alfred Schütz [27],

13 The importance of addressing the individual needs of each patient is particularly evident in a qualitative empirical study conducted by a research group at the University of Basel. The study explored the reasons behind the wish to die expressed by oncology patients receiving palliative care at various stages. A key finding is that this wish is profoundly shaped by the patient’s personal history, core values, and relational context—especially their connections with family members. The motivations are largely spiritual and existential, rather than primarily driven by physical suffering. These findings highlight not only the relevance of the concept of *total pain* [34], but also the need for care environments that support and integrate the familial and relational dimensions of the patient's experience [32].

14 Such attention to the existential and bodily dimensions of care resonates with the phenomenological works of Svenaeus [35], who emphasizes how illness can transform patients’ being-in-the-world and thus their habitual existence, as also argued by Kay Toombs, and Carel [36], who highlights the centrality of vulnerability and embodiment in understanding human flourishing. Together, these works further demonstrate how phenomenological insights can enrich ethical reflection and inform the design of care environments that are responsive to both practical and existential needs.
